# Parvalbumin-Expressing GABAergic Neurons in Mouse Barrel Cortex Contribute to Gating a Goal-Directed Sensorimotor Transformation

**DOI:** 10.1016/j.celrep.2016.03.063

**Published:** 2016-04-14

**Authors:** Shankar Sachidhanandam, B. Semihcan Sermet, Carl C.H. Petersen

**Affiliations:** 1 Laboratory of Sensory Processing, Faculty of Life Sciences, Brain Mind Institute, École Polytechnique Fédérale de Lausanne (EPFL), 1015 Lausanne, Switzerland

## Abstract

Sensory processing in neocortex is primarily driven by glutamatergic excitation, which is counterbalanced by GABAergic inhibition, mediated by a diversity of largely local inhibitory interneurons. Here, we trained mice to lick a reward spout in response to whisker deflection, and we recorded from genetically defined GABAergic inhibitory neurons in layer 2/3 of the primary somatosensory barrel cortex. Parvalbumin-expressing (PV), vasoactive intestinal peptide-expressing (VIP), and somatostatin-expressing (SST) neurons displayed distinct action potential firing dynamics during task performance. Whereas SST neurons fired at low rates, both PV and VIP neurons fired at high rates both spontaneously and in response to whisker stimulation. After an initial outcome-invariant early sensory response, PV neurons had lower firing rates in hit trials compared to miss trials. Optogenetic inhibition of PV neurons during this time period enhanced behavioral performance. Hence, PV neuron activity might contribute causally to gating the sensorimotor transformation of a whisker sensory stimulus into licking motor output.

## Highlights

• Cell-type-specific firing of neocortical GABAergic neurons during task performance
• Parvalbumin-expressing GABAergic neurons fire at lower rates in hit versus miss trials
• Optogenetic inhibition of parvalbumin-expressing neurons increases hit rates
• These neurons thus contribute to transformation of stimulus into action

## Graphical Abstract

**Figure:**
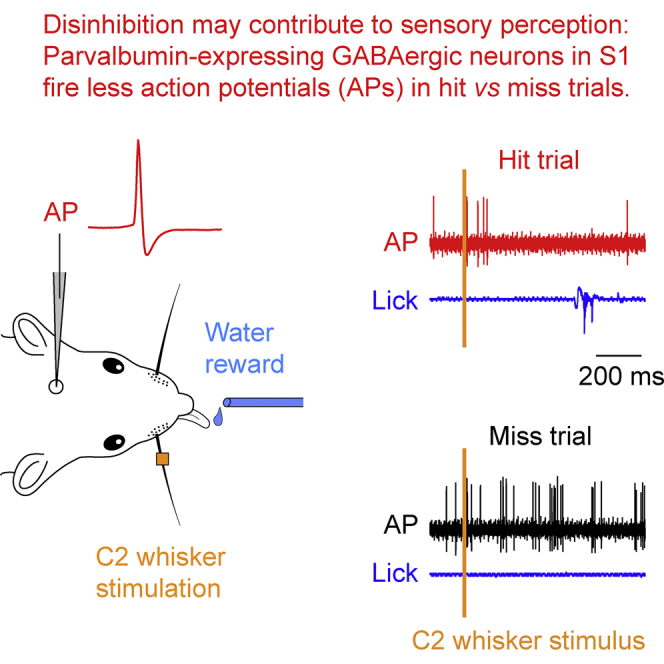

## Teaser

Sachidhanandam et al. report that parvalbumin-expressing GABAergic neurons in mouse barrel cortex fire fewer action potentials in hit trials versus miss trials during a whisker-dependent detection task. Optogenetic inhibition of these neurons enhances behavioral performance. Parvalbumin-expressing neurons thus contribute to gating sensorimotor transformation of whisker stimulus into goal-directed motor output.

## Introduction

The neocortex has a diversity of GABAergic inhibitory neurons that differ in electrophysiological properties, structural features, synaptic connectivity, gene expression, and developmental origin (Ascoli et al., 2008). Based on the expression of largely non-overlapping molecular markers, these neurons can be classified into three groups: parvalbumin expressing (PV), somatostatin expressing (SST), and 5HT_3A_ receptor expressing, which includes neurons expressing vasoactive intestinal peptide (VIP) (Lee et al., 2010). Through targeting specific cellular compartments of excitatory neurons, as well as by inhibiting other GABAergic neurons, these genetically defined inhibitory neuron populations are likely to differentially control distinct aspects of cortical function (Isaacson and Scanziani, 2011, Kepecs and Fishell, 2014). Previous studies have found that different GABAergic neuron subtypes exhibit distinct and diverse activities during different behavioral states (Gentet et al., 2012, Lee et al., 2013, Polack et al., 2013, Schneider et al., 2014, Fu et al., 2014) and different learned behaviors (Lee et al., 2012, Kvitsiani et al., 2013, Pi et al., 2013, Zhang et al., 2014, Pinto and Dan, 2015).

Here we investigated the firing patterns of genetically defined populations of GABAergic neurons in layer 2/3 of primary somatosensory barrel cortex (S1) during a barrel cortex-dependent task in which thirsty mice need to convert sensory information evoked by a whisker deflection into a goal-directed motor output of licking a spout for water reward (Sachidhanandam et al., 2013, Sippy et al., 2015). In a previous study using the same detection task (Sachidhanandam et al., 2013), we reported that GABAergic neurons in layer 2/3 of S1 fire at high rates, but the differential contributions of distinct subtypes of GABAergic neurons during task performance were not investigated. In this study, we therefore recorded the activity of PV, VIP, and SST neurons during the detection task, finding that both PV and VIP neurons fired at high rates during task performance, with PV neurons firing less action potentials (APs) in hit trials compared to miss trials. Our results suggest that PV neurons in S1 might contribute to gating the goal-directed sensorimotor transformation of sensory stimuli into licking motor output.

## Results

**Figure 1:**
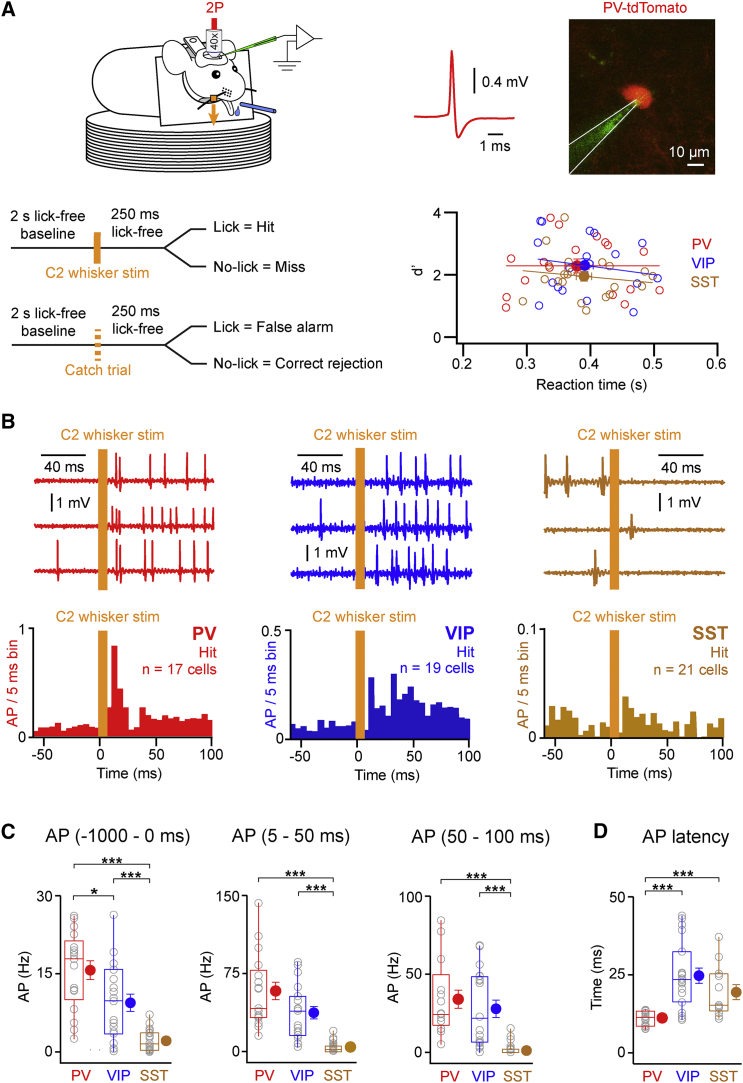
Cell-Type-Specific AP Firing of GABAergic Neurons in Hit Trials during a Whisker Detection Task (A) Top left: setup for two-photon (2P) guided targeting of juxtasomal recordings during the head-fixed whisker deflection detection task. Top right: 2P view shows a PV neuron expressing tdTomato (red) targeted for juxtasomal recording with a pipette containing Alexa-488 (green), together with an example spike recorded from the PV neuron. Bottom left: schematic shows trial types and outcomes of the behavioral task. Bottom right: plot of d′ against reaction time for the different recordings indicates no difference in performance among the different genotypes of mice (each point represents an individual recording from a specific genetically labeled neuron, as indicated by color coding). Lines indicate best fits for PV, VIP, and SST data (linear correlation for PV: *r* = −0.0004, p = 0.99, t test, n = 17 cells; for VIP: *r* = −0.18, p = 0.46, t test, n = 19 cells; for SST: *r* = −0.16, p = 0.50, t test, n = 21 cells). (B) Example hit trials and grand average peri-stimulus time histogram (PSTH) of AP discharge of PV, VIP, and SST neurons for hit trials in response to C2 whisker stimulus. Note the difference in scale for the number of APs discharged per 5-ms bin between the different types of GABAergic neurons. (C) Baseline (1 s) and post-whisker stimulus (5–50 ms and 50–100 ms) AP discharge rates are shown. (D) AP latencies of the first spike after whisker stimulus for PV, VIP, and SST neurons are shown. Open circles represent individual cells. Filled circles with error bars represent group averages shown as mean ± SEM. Boxplots represent the median, the 25^th^ and 75^th^ percentiles in the boxes, with the side bars representing the 5^th^ and 95^th^ percentiles of the distribution. Statistical significance is indicated as follows: ^∗^p < 0.05 and ^∗∗∗^p < 0.005. See also Figure S1.

Under visual control offered by a two-photon microscope, we targeted juxtasomal recordings to fluorescently labeled neurons in PV-Cre (Hippenmeyer et al., 2005), VIP-Cre (Taniguchi et al., 2011), and SST-Cre (Taniguchi et al., 2011) mice crossed with tdTomato-expressing Cre-reporter mice (Madisen et al., 2010) (Figure 1A). In some experiments, SST neurons were recorded in GIN-GFP mice (Oliva et al., 2000, Gentet et al., 2012). To separate the sensory response from the motor report, we analyzed hit trials with reaction times of more than 250 ms (Figure 1A). An analysis of all the trials (including both short and long reaction times) revealed that our results were invariant to this selection procedure (Figure S1).

We observed that baseline firing (quantified over 1 s before whisker stimulus) differed strongly across cell types with PV neurons having the highest AP firing rates, and both PV and VIP neurons firing more than SST cells (mean ± SEM, median: PV 15.7 ± 1.8 Hz, 17.8 Hz, n = 17 cells, n = 9 mice; VIP 9.4 ± 1.7 Hz, 9.8 Hz, n = 19 cells, n = 6 mice; SST 2.1 ± 0.4 Hz, 1.6 Hz, n = 21 cells, n = 6 mice; Figures 1B and 1C). Deflection of the C2 whisker evoked a rapid cell-type-specific increase in firing (quantified from 5 to 50 ms after stimulus) with both PV and VIP neurons firing at significantly higher rates compared to SST neurons (mean ± SEM, median: PV 57.9 ± 8.5 Hz, 41.3 Hz, n = 17 cells, n = 9 mice; VIP 37.0 ± 6.0 Hz, 38.9 Hz, n = 19 cells, n = 6 mice; SST 3.7 ± 1.1 Hz, 2.0 Hz, n = 21 cells, n = 6 mice; Figures 1B and 1C). The firing rates of PV and VIP neurons, but not SST neurons, increased significantly compared to their respective baseline rates (p = 1.5 × 10^−5^ for PV; p = 7.6 × 10^−6^ for VIP; p = 0.84 for SST).

Both PV and VIP neurons continued to fire at high rates for a prolonged period after this initial 50-ms period of sensory processing, which was particularly prominent for VIP neurons (Figure 1B). Quantified from 50 to 100 ms post-stimulus and compared to 5–50 ms after stimulus, the firing rate did not drop significantly for VIP neurons (p = 0.11, n = 19 cells, n = 6 mice), unlike PV cells that significantly decreased in firing rates (p = 1.5 × 10^−5^, n = 17 cells, n = 9 mice). Selecting for trials with firing in the first 50 ms post-stimulus and computing means only for the cells with firing in that period, PV cells fired at shorter latencies following whisker stimulus compared to the other cell types (mean ± SEM, median: PV 11.0 ± 0.5 ms, 11.3 ms, n = 17 cells, n = 9 mice; VIP 24.8 ± 2.4 ms, 23.5 ms, n = 19 cells, n = 6 mice; SST 19.6 ± 2.4 ms, 15.3 ms, n = 12 cells, n = 5 mice; Figure 1D). The shorter latency of PV neuron firing likely results from glutamatergic synaptic input onto PV neurons from both thalamocortical and intracortical sources being larger and faster than onto other cell types (Gibson et al., 1999, Mateo et al., 2011). Hence the different GABAergic neuron subtypes exhibited distinct response profiles during task execution, with both PV and VIP neurons firing APs at high rates during cortical processing of the whisker stimulus, thus being likely to contribute importantly to regulating S1 neuronal network activity by inhibiting postsynaptic neurons.

We previously reported that whisker deflection evokes a depolarizing response with two distinct components in S1 excitatory neurons during the same detection task (Sachidhanandam et al., 2013) as follows: an early response (5–50 ms post-stimulus) that is invariant with respect to trial outcome and a later secondary response (50–250 ms post-stimulus), which is enhanced in hit trials (Figure S2). The secondary response therefore correlates with behavioral report of perceived stimuli. Optogenetic inactivation of S1 furthermore shows that the late secondary activity in excitatory neurons causally contributes to perceptual report (Sachidhanandam et al., 2013).

**Figure 2:**
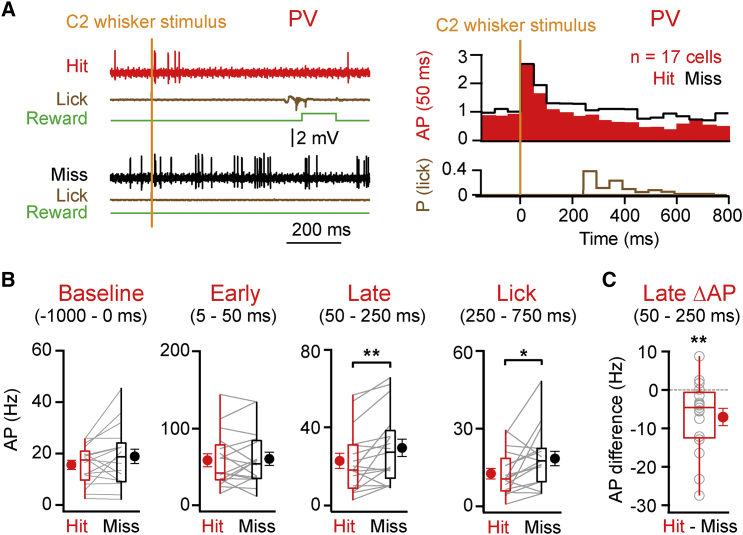
PV-Expressing GABAergic Neurons Fire Fewer APs in Hit Trials Compared to Miss Trials (A) Left: example hit and miss trials during a recording from a PV neuron. Right: grand average PSTH shows PV neurons recorded during the detection task, analyzed separately for hit (red) and miss (black) trials, together with a histogram of the first lick reaction time (brown). (B) AP discharge rates of PV neurons before whisker stimulus (−1,000–0 ms), during early sensory processing (5–50 ms post-whisker stimulus), during the late period (50–250 ms), and during licking (250–750 ms) in hit and miss trials are shown. (C) AP discharge rate difference between hit and miss trials of PV neurons during the late period is shown. Lines and open circles represent individual cells. Filled circles with error bars represent group averages shown as mean ± SEM. Boxplots represent the median, the 25^th^ and 75^th^ percentiles in the boxes, with the side bars representing the fifth and 95^th^ percentiles of the distribution. Statistical significance is indicated as follows: ^∗^p < 0.05 and ^∗∗^p < 0.01. See also Figure S2.

Here we found that PV cells displayed similar increases in AP firing rates in the early evoked sensory response in both hit and miss trials (mean ± SEM, median quantified 5–50 ms post-stimulus: hit 57.9 ± 8.5 Hz, 41.3 Hz; miss 59.7 ± 8.6 Hz, 53.3 Hz; p = 0.78; n = 17 cells, n = 9 mice; Figures 2A and 2B), indicating that these neurons do not discriminate trial outcome during this early period. However, during the later secondary response, PV cells displayed firing rates that were higher in misses compared to hits (mean ± SEM, median quantified 50–250 ms post-stimulus: hit 22.6 ± 3.9 Hz, 18.0 Hz; miss 29.3 ± 4.5 Hz, 27.1 Hz; p = 0.0056; n = 17 cells, n = 9 mice; Figures 2A and 2B). Over the 200-ms period quantified, this equals 3.6 spikes in hits compared to 5.4 spikes in misses (computed from the medians). During the late period, PV neurons fired more in miss trials (difference in AP discharge rate between hits and misses, mean ± SEM: −6.8 ± 2.3 Hz; median: −4.6 Hz; p = 0.0075), and 13 of 17 PV cells had higher AP discharge rates on miss trials compared to hits (Figure 2C). These findings suggest that a reduction of PV neuron activity in S1 during the late period of hit trials (compared to miss trials) might contribute to allowing nearby excitatory cells to increase their firing rate on hit trials (Figure S2; Sachidhanandam et al., 2013).

**Figure 3:**
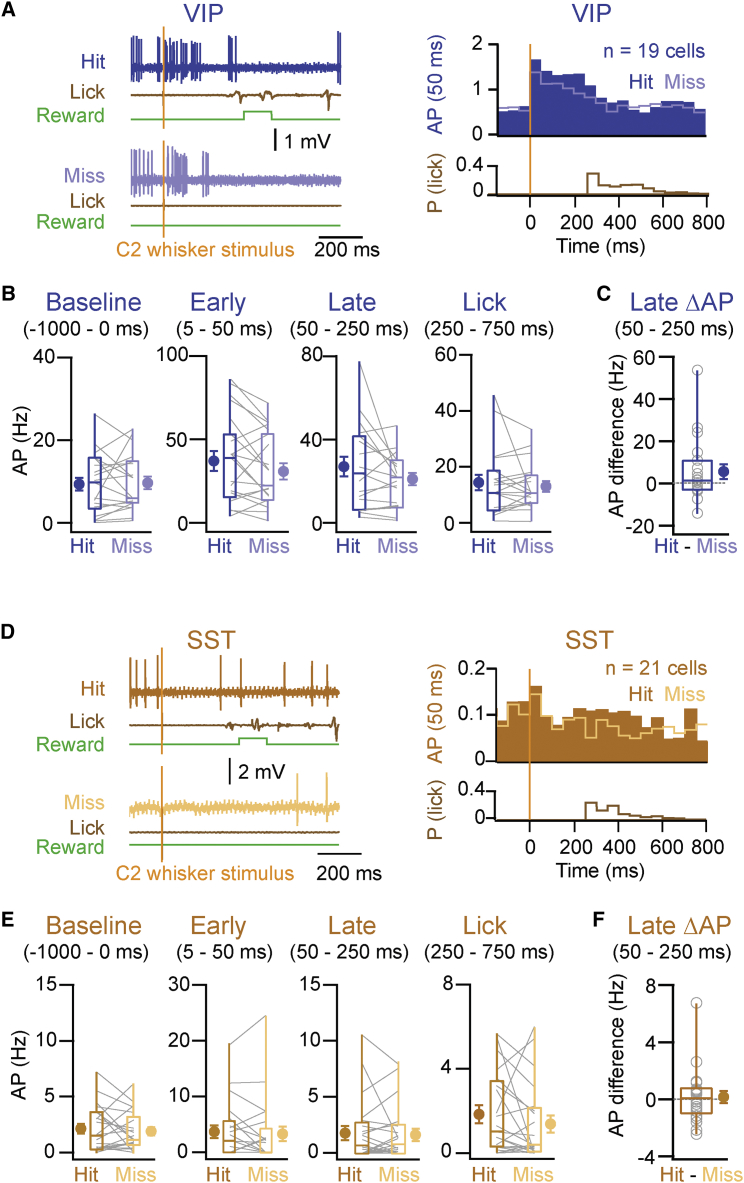
Comparison of Hit and Miss Trials for VIP- and SST-Expressing GABAergic Neurons (A) Left: example hit and miss trials during a recording from a VIP neuron. Right: grand average PSTH shows VIP neurons during the detection task, in hit and miss trials together, with a histogram of the first lick reaction time. (B) AP discharge rates of VIP neurons before whisker stimulus (−1,000–0 ms), during early sensory processing (5–50 ms post-whisker stimulus), during the late period (50–250 ms), and during licking (250–750 ms) in hit and miss trials. (C) AP discharge rate difference between hit and miss trials of VIP neurons during the late period is shown. (D–F) Same as (A)–(C) are shown, but for SST neurons. Lines and open circles represent individual cells. Filled circles with error bars represent group averages shown as mean ± SEM. Boxplots represent the median, the 25^th^ and 75^th^ percentiles in the boxes, with the side bars representing the fifth and 95^th^ percentiles of the distribution. No statistically significant differences were found comparing hit and miss trials.

We also analyzed hit versus miss trials of recordings from VIP and SST neurons, both of which have been implicated in disinhibitory neuronal circuits (Lee et al., 2013, Pfeffer et al., 2013, Xu et al., 2013). However, perhaps because of our small dataset, we did not find statistically significant differences comparing hit and miss trials with respect to AP firing in VIP and SST cells (Figure 3). The early evoked sensory response quantified from 5 to 50 ms did not differ significantly across hit and miss trials for VIP cells (p = 0.13, n = 19 cells, n = 6 mice; Figures 3A and 3B) or for SST cells (p = 0.53, n = 21 cells, n = 6 mice; Figures 3D and 3E). Similarly, AP firing during the late secondary period (50–250 ms) did not differ significantly comparing hit and miss trials for VIP (p = 0.21, n = 19 cells, n = 6 mice; Figures 3A and 3B) or for SST neurons (p = 0.88, n = 21 cells, n = 6 mice; Figures 3D and 3E). The AP rate difference between hit and miss trials during this period also was not significant (VIP p = 0.20, n = 19 cells, n = 6 mice; SST p = 0.87, n = 21 cells, n = 6 mice; Figures 3C and 3F). Thus, analyzed as groups in our limited dataset, neither VIP nor SST neurons displayed significant task outcome-related differences in AP firing.

**Figure 4:**
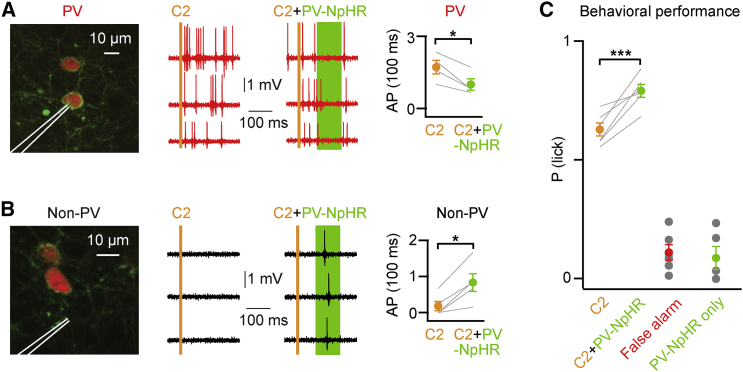
Optogenetic Inhibition of PV-Expressing GABAergic Neurons in S1 Can Enhance Behavioral Performance (A) Left: a 2P image showing a juxtasomal recording electrode targeted to a PV neuron expressing eNpHR3.0. Center: example traces of a PV neuron expressing NpHR show AP discharge suppression upon application of yellow light (80–180 ms post-whisker stimulus, green shading) coupled with C2 whisker stimulus. Right: group statistics of AP discharge suppression in PV-NpHR neurons quantified 80–180 ms post-whisker stimulus are shown. (B) Left: a 2P image showing a juxtasomal recording electrode targeted to a non-PV (presumed excitatory) neuron. Center: example traces of a non-PV neuron show enhanced AP discharge upon application of yellow light (80–180 ms post-whisker stimulus) coupled with C2 whisker stimulus. Right: group statistics of AP discharge in these non-PV neurons quantified 80–180 ms post-whisker stimulus are shown. (C) Yellow light coupled with C2 whisker stimulus enhanced performance over C2 stimulus alone in PV-NpHR mice. Yellow light delivery alone did not result in an increase in false alarm rates. Gray lines represent individual cells in (A) and (B). Gray lines and gray circles represent individual mice in (C). Color-coded circles with error bars represent group averages shown as mean ± SEM. Statistical significance is indicated as follows: ^∗^p < 0.05 and ^∗∗∗^p < 0.005. See also Figure S3.

Our data suggest that PV neurons in S1 might modulate the transformation of sensory information into motor output on a trial-by-trial basis during this detection task. Elevated AP firing rates in PV neurons during the late period correlated with miss trials. Consistent with this result, in a previous study (Sachidhanandam et al., 2013) we found that behavioral performance could be reduced by optogenetically stimulating PV neurons expressing ChR2 during the late secondary response period. Conversely, a reduction in AP firing rate (in hit trials compared to misses) in PV neurons during this period was associated with hit trials, and, here, we therefore optogenetically tested whether direct inhibition of PV neurons might be able to enhance behavioral report of the whisker stimulus (Figure 4). We injected a Cre-dependent adeno-associated virus into S1 of PV-Cre mice to express the light-activated chloride pump halorhodopsin (eNpHR3.0) (Gradinaru et al., 2010) in PV neurons (Figure S3). Targeting recordings to PV-NpHR-expressing neurons, we found that yellow light delivered 80–180 ms post-stimulus, timed to coincide with the onset of the late period (Sachidhanandam et al., 2013), significantly reduced AP firing in PV cells during light delivery (mean ± SEM quantified 80–180 ms post-stimulus: C2 whisker stimulus alone evoked 1.7 ± 0.3 spikes; C2 whisker stimulus together with PV-NpHR evoked 1.0 ± 0.2 spikes; Student’s paired t test p = 0.026; n = 4 cells, n = 4 mice; Figure 4A). Quantified during the entire late phase, as for the hit versus miss comparison (50–250 ms post-stimulus), PV-NpHR neurons fired at 17.0 ± 5.2 Hz in trials without yellow light, and they fired at 13.3 ± 3.3 Hz in trials with yellow light (n = 4 cells, n = 4 mice). The activity of nearby non-fluorescently labeled neurons, presumably excitatory neurons, was enhanced (mean ± SEM quantified 80–180 ms post-stimulus: C2 whisker stimulus alone evoked 0.2 ± 0.1 spikes; C2 whisker stimulus together with PV-NpHR evoked 0.8 ± 0.2 spikes; Student’s paired t test p = 0.011; n = 5 cells, n = 4 mice; Figure 4B). Mice showed improved performance in trials where we coupled a yellow light flash with whisker stimulation (80–180 ms post-stimulus) without a change in false alarm rates (mean ± SEM: C2 whisker hit rate 63% ± 3%; C2 whisker with PV-NpHR hit rate 79% ± 3%; false alarm rate 11% ± 3%; false alarm rate with PV-NpHR 8% ± 4%; n = 6 mice; Student’s paired t test p = 0.003 for C2 whisker versus C2 whisker with PV-NpHR; Figure 4C). These results indicate that PV cell activity in S1 during the secondary late response period can contribute to modulating behavioral outcome.

## Discussion

We found that different GABAergic neuron subtypes in S1 have distinct response profiles during whisker sensory perception when engaged in a task. Whereas SST neurons fired at low rates, both PV and VIP neurons responded strongly to whisker stimulus, with PV neurons firing at shorter latencies. PV neurons fired significantly more in miss than in hit trials during a period after the early sensory response and before licking. PV neurons might therefore contribute to gating sensorimotor transformation after initial sensory processing. The optogenetic inhibition of PV cell activity during the late phase enhanced behavioral performance consistent with the hypothesis that PV cell activity might be causally involved in determining task hit/miss outcome, presumably exerting its effect through controlling the activity of nearby excitatory neurons.

It is important to note the limitations of the optogenetic manipulations carried out in this study in terms of how closely they match the physiological modulation that we found comparing hit and miss trials. Specifically, we did not achieve layer specificity with our viral expression of NpHR, and in our experiments we therefore likely also inhibited PV neurons in deeper layers, whereas we do not currently know their physiological firing patterns. Equally, the timing and degree of optogenetic inhibition of the PV neurons probably only roughly mimicked the physiological modulation comparing hit and miss trials. The optogenetic inhibition produced a PV firing rate modulation of 0.78 (ratio of mean firing rate: light/no light), which is comparable to the hit versus miss PV firing rate modulation of 0.77 (ratio of mean firing rate: hit/miss) during the 50- to 250-ms window in the detection task. In future experiments, it will be of interest to more precisely control the optogenetic inhibition of PV neurons, attempting to match their behavioral firing rate modulation for each individually recorded PV neuron.

While the mechanisms controlling differential hit versus miss activity in PV cells for this behavioral paradigm remain to be identified, it is tempting to speculate the possible involvement of cholinergic input acting via layer 1 inhibitory neurons (Letzkus et al., 2011) or direct long-range GABAergic inhibition from basal forebrain known to specifically target PV neurons (Freund and Meskenaite, 1992, Kim et al., 2015). A recent study showed that top-down cortical input from secondary motor cortex drove a late response in excitatory neurons (Manita et al., 2015). Hence, it could be possible that long-range inputs (for example from M1, S2, thalamus, basal ganglia, or neuromodulatory inputs) could differentially activate PV neurons during the task or indirectly modulate their activity via disinhibitory circuits.

In our limited dataset, we did not find significant differences in AP discharge comparing hit and miss trials for VIP and SST neurons. Previous studies found that VIP neurons showed enhanced activity during reinforcement signals (reward and punishment) (Pi et al., 2013), as well as during motor/whisking activity (Lee et al., 2013). VIP and SST neurons in different brain areas and cortical layers might have different activity patterns during the diverse behaviors investigated to date. That we did not find significant differences in AP discharge comparing hits and misses for VIP and SST neurons in part may relate to the small number of recorded neurons (n = 19 VIP cells; n = 21 SST cells), giving rise to low statistical power, or an overall small effect size for these types of neurons in S1 layer 2/3 for our specific detection task. Equally, each of the groups of neurons expressing either VIP or SST is likely to contain distinct subgroups (McGarry et al., 2010, Prönneke et al., 2015), which could show different outcome-related activity patterns. In future experiments it will therefore likely be important to further refine the definition of GABAergic cell types and record their activity during diverse behaviors.

## Experimental Procedures

All experimental procedures were approved by the Swiss Federal Veterinary Office.

### Animals and Surgery

PV-Cre (Hippenmeyer et al., 2005), VIP-Cre (Taniguchi et al., 2011), and SST-Cre (Taniguchi et al., 2011) mice were crossed to LoxP-STOP-LoxP-tdTomato Cre-reporter mice (Madisen et al., 2010). In some experiments, SST neurons were recorded in GIN-GFP mice (n = 8 SST cells were recorded in two GIN-GFP mice and n = 13 SST cells were recorded in four SST-Cre mice) (Gentet et al., 2012). Mice were implanted with a metal head restraint post at 4–9 weeks after birth under isoflurane anesthesia. All whiskers were trimmed except for the C2 whiskers on either side. Intrinsic signal optical imaging was carried out to locate the C2 barrel column in the left hemisphere.

### Behavioral Training

The behavioral training was carried out as previously described (Sachidhanandam et al., 2013). Briefly, water-restricted mice were taught to associate a 1-ms magnetic pulse applied to iron particles attached to the right C2 whisker with water availability, delivered via a reward spout. A drop of water was delivered if they licked within the reward time window (0–750 ms post-whisker stimulus). Whisker stimuli were delivered without preceding cues at random inter-stimulus intervals ranging from 2 to 8 s. Catch trials (no whisker stimulus) were randomly interleaved with whisker stimulus trials to obtain the false alarm rates. A lick-free 2-s period was imposed before trial initiation. Behavioral control and behavioral data collection were carried out with custom-written computer routines using an ITC18 (Instrutech) interfaced through IgorPro (Wavemetrics). Once the mice achieved a consistent hit rate above 80% and false alarms lower than 30%, they were considered well trained and they subsequently were used for electrophysiological recordings. For the optogenetic manipulations, mice were trained until they achieved hit rates between 60% and 80%, so as avoid to a ceiling effect.

### Electrophysiology

Recording electrodes were targeted to the left C2 barrel column identified through intrinsic signal optical imaging. All recordings were obtained from layer 2/3 using standard glass patch-clamp electrodes with resistance of ∼5 MΩ. The pipettes were filled with Ringer’s solution containing the following (in mM): 135 NaCl, 5 KCl, 5 HEPES, 1.8 CaCl_2_, 1 MgCl_2_, and 10 μM Alexa-594 (for recording GFP neurons) or Alexa-488 (for recording tdTomato neurons). All recordings were carried out under visual control with a custom-built two-photon microscope. A pulsed laser (MaiTai HP) focused 920-nm light into the cortex using a 40× 0.9 numerical aperture (NA) objective (Olympus), and fluorescence was detected on red and green channels (red 607 ± 35 nm and green 510 ± 42 nm) using photomultiplier tubes (PMTs, Hamamatsu). All electrophysiological measurements were made with a Multiclamp 700B amplifier (Molecular Devices) filtered at 10 kHz and digitized at 20 kHz by an ITC-18 under the control of IgorPro. Offline filtering (300 Hz to 1 kHz) was performed to isolate spikes. Because of the technical difficulty of maintaining long-lasting recordings from GABAergic neurons, we only recorded from a limited number of trials for each cell. On average we recorded 14 hit versus 13 miss trials for PV neurons; 15 hit versus 12 miss trials for VIP neurons; and 18 hit versus 16 miss trials for SST neurons.

### Optogenetics

PV-Cre mice crossed to LoxP-STOP-LoxP-tdTomato reporter mice (5-week-old males) were injected with AAV-DIO-eNpHR3.0-YFP in the C2 barrel column, identified through intrinsic signal optical imaging. A single injection of ∼350 nl was carried out at a depth of ∼300 μm below the pia through a ∼0.5-mm craniotomy.

Mice expressing eNpHR3.0 in PV neurons were trained in the whisker stimulus detection task, as described above, except now in an environment with ambient yellow light. On the day of optogenetic PV inactivation, both NpHR stimulus trials coupled with the whisker stimulus and uncoupled NpHR stimulus trials were randomly interleaved with the whisker stimulus and catch trials. The light stimulus consisted of a continuous yellow light pulse and was applied from 80 to 180 ms post-whisker stimulus, matching the rising phase of the excitatory postsynaptic potentials (EPSPs) during the late period in excitatory neurons (Sachidhanandam et al., 2013). Light stimuli were delivered to the barrel cortex by a 7-mW, 591-nm yellow light-emitting diode (LED, Luxeon, Philips) focused through the 40× 0.9 NA objective (Olympus). At the end of the experiment, mice were anesthetized, perfused with 4% paraformaldehyde (PFA), and their brains removed. To visualize the eNpHR3.0-YFP injection sites, 60-μm coronal sections containing barrel cortex were cut on a vibratome (Leica VT1000S). To enhance the NpHR3.0-YFP signal, sections were stained with a primary antibody against GFP (rabbit, 1:2,000, Abcam Ab290; 24 hr) followed by secondary goat anti-rabbit antibody coupled to Alexa 488 (1:200, Invitrogen; 2 hr). Slices were then stained with 2.5 μM DAPI for 10 min and mounted on glass slides with DABCO. Images were obtained using an epifluorescence microscope (Olympus Slide Scanner VS120-L100) through a 4×/0.16 NA air objective (Figure S3).

### Statistical Analysis

All values are presented as mean ± SEM and/or medians (stated in the text). Boxplots represent the median, the 25^th^ and 75^th^ percentiles in the boxes, and the side bars represent the fifth and 95^th^ percentiles of the distribution. Statistical testing was carried out in IgorPro, MATLAB, and Microsoft Excel. The Anderson-Darling test was done on all the data to test for normality. We used Student’s two-tailed paired or unpaired t test for parametric data, and we used the Wilcoxon signed-rank paired test and the Wilcoxon-Mann-Whitney test for paired and unpaired non-parametric data. All tests were two sided. The sensitivity index d′ (d prime) from signal detection theory was computed as d′ = z (hit rate) − z (false alarm rate), with the *Z* scores computed in Excel using the function NORMSINV.

## Author Contributions

S.S. and C.C.H.P. designed the project and wrote the manuscript. S.S. carried out all experiments and data analyses. B.S.S. carried out histology and commented on the manuscript.

## Supplemental Information

Document S1. Figures S1–S3

Document S2. Article plus Supplemental Information
