# Supplementary material for: Parvalbumin-Expressing GABAergic Neurons in Mouse Barrel Cortex Contribute to Gating a Goal-Directed Sensorimotor Transformation
Source: Cell Rep. 2016 Apr 14;15(4):700–6. doi: 10.1016/j.celrep.2016.03.063 (PMC4850419; doi:10.1016/j.celrep.2016.03.063)
Supplement: Document S1. Figures S1–S3 [file mmc1.pdf]

Cell Reports, Volume *15*

Supplemental Information

Parvalbumin-Expressing GABAergic Neurons in Mouse Barrel Cortex Contribute to Gating a Goal-Directed Sensorimotor Transformation

Shankar Sachidhanandam, B. Semihcan Sermet, and Carl C.H. Petersen

Supplemental Information

Parvalbumin-expressing GABAergic neurons in mouse barrel cortex contribute to gating a goal-directed sensorimotor transformation

Shankar Sachidhanandam, B. Semihcan Sermet
and Carl C.H. Petersen

Supplemental information consists of:

Supplemental Figure S1 (related to Figure 1)
Supplemental Figure S2 (related to Figure 2)
Supplemental Figure S3 (related to Figure 4)

Supplemental Figure S1

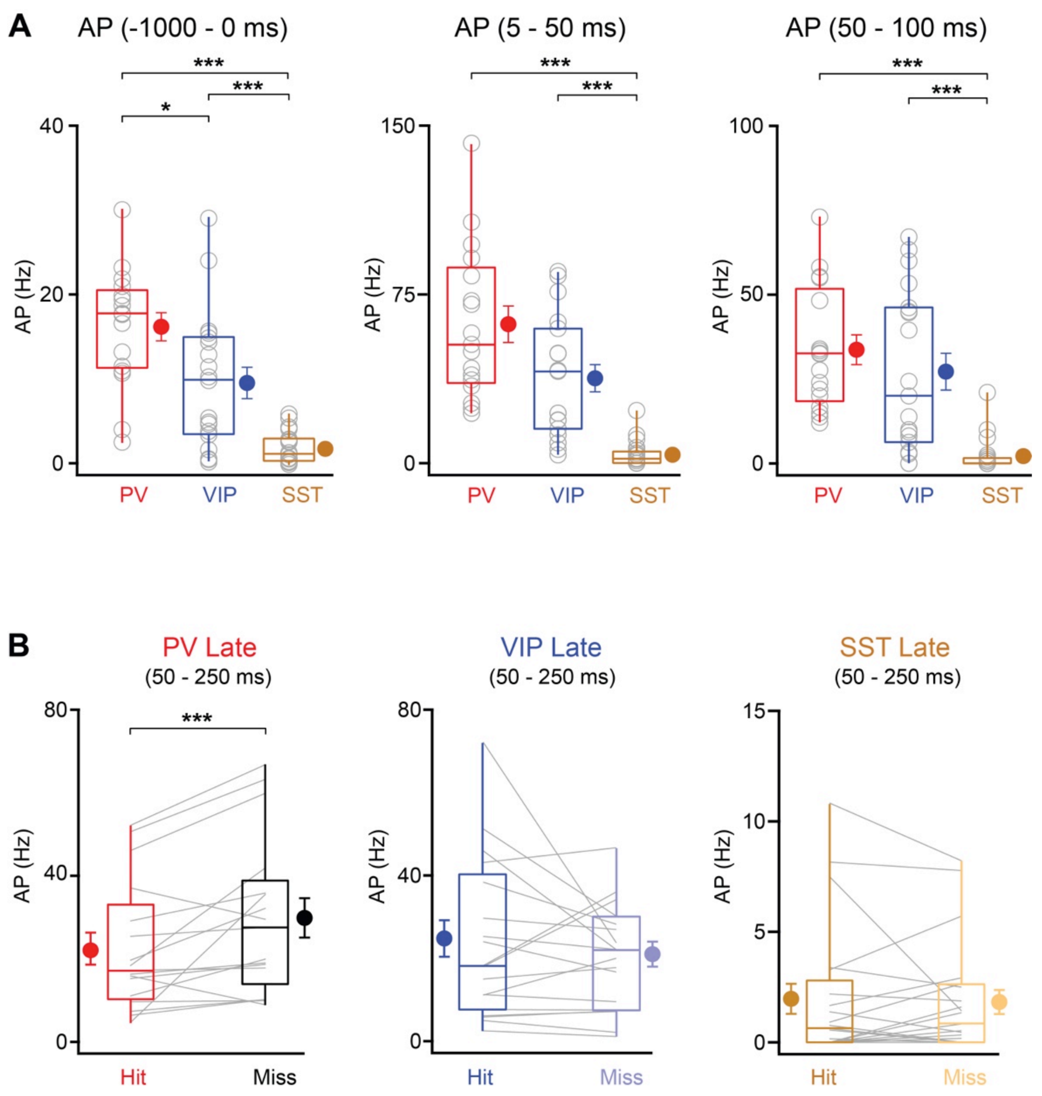

Figure S1. Baseline, early sensory responses and late phase differences were not influenced by selection of reaction times (related to Figure 1).

(A) Differences in AP rates during the baseline (-1000 – 0 ms) and post whisker stimulus (5 – 50 ms, and 50 – 100 ms) between PV, VIP and SST neurons were unaffected when all trials were included in the analysis (including hit trials with licks earlier and later than 250 ms post-whisker stimulus) (see Figure 1C).

(B) PV neurons showed trial outcome related differences in AP rates during the late phase (50 – 250 ms) when all trials were included (including both short and long reaction times), comparable to the late phase without early licks (see Figure 2B). VIP and SST neurons showed no trial outcome related differences during the late period,

when all trials were included, similar to the late phase without early licks (see Figures 3B and 3E).

Lines and open circles represent individual cells. Filled circles with error bars represent group averages shown as mean ± sem. Box plots represent the median, the 25th and 75th percentiles in the boxes, whereas the side bars represent the 5th and 95th percentiles of the distribution. Statistical significance is indicated by * for $P < 0.05$ and *** for $P < 0.005$.

Supplemental Figure S2

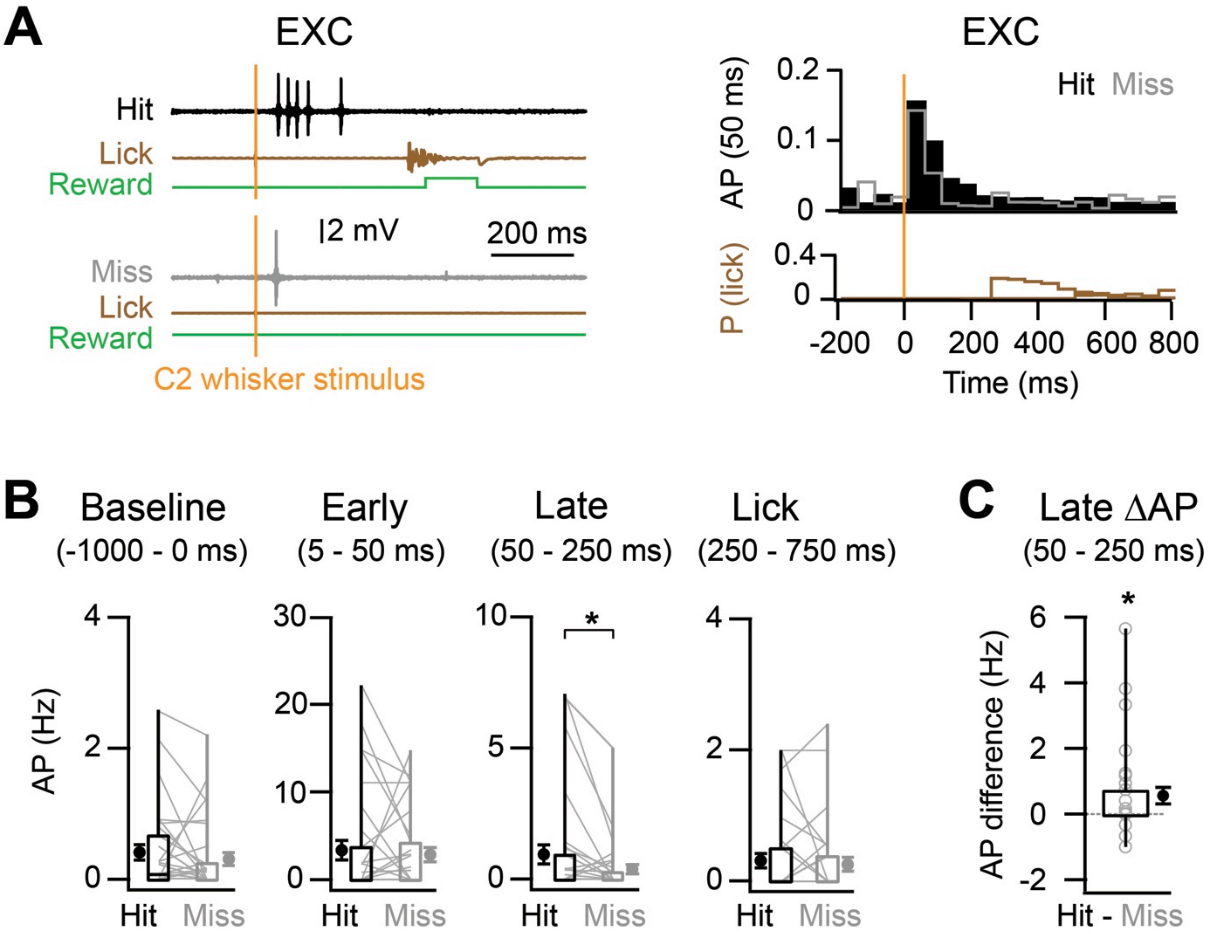

Figure S2. Excitatory neurons display trial outcome dependent responses (related to Figure 2).

(A) Left: example hit and miss trials from an excitatory (EXC) neuron. Right: grand average PSTH of n = 31 EXC neurons during the task in hit and miss trials. Note the enhanced AP discharge during the late period (50 – 250 ms post whisker stimulus) in hit compared to miss trials, before behavioral report via licking.

(B) AP discharge rates of EXC neurons, before whisker stimulus (-1000 – 0 ms) and during early sensory processing (5 – 50 ms post whisker stimulus) were indifferent to behavioral outcome. AP rates were significantly enhanced during the late period (50 – 250 ms) in hit trials, but not during licking (250 – 750 ms).

(C) AP discharge rate difference between hit and miss trials of EXC neurons during the late period (signed rank test, P = 0.02, n = 31).

Lines and open circles represent individual cells. Filled circles with error bars represent group averages shown as mean ± sem. Box plots represent the median, the 25th and 75th percentiles in the boxes, whereas the side bars represent the 5th and 95th percentiles of the distribution. Statistical significance is indicated by * for $P < 0.05$.

All data from EXC neurons are reanalyzed from Sachidhanandam et al. (2013) to include only trials that are lick-free between 0 and 250 ms post whisker stimulus.

Supplemental Figure S3

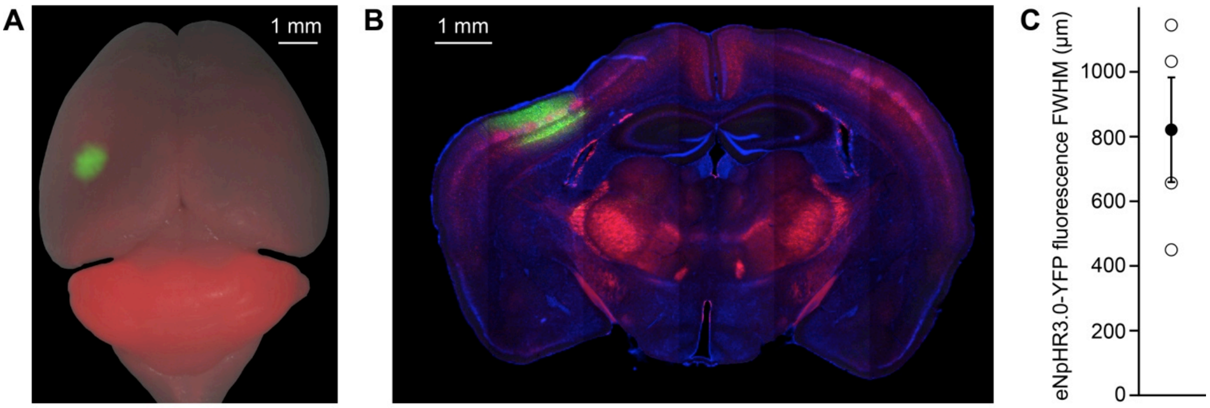

Figure S3. Expression of eNpHR3.0-YFP in PV-expressing GABAergic neurons (related to Figure 4).

(A) eNpHR3.0-YFP (green) was expressed in PV-expressing neurons by injecting a Cre-dependent AAV vector into the C2 barrel column of S1 barrel cortex in PV-Cre mice, which were then trained to detect C2 whisker stimuli. After training and electrophysiological recordings, the mice were fixed with PFA and the fluorescence from the whole-brain imaged. Red fluorescence shows tdTomato expression in PV neurons (PV-Cre mice were crossed to LoxP-STOP-LoxP-tdTomato reporter mice).

(B) Coronal section through the center of the eNpHR3.0-YFP expression site (green) from the same mouse as shown in panel A. eNpHR3.0-YFP fluorescence was enhanced through antibody staining against GFP. Red fluorescence shows tdTomato expression in PV neurons, with prominent red fluorescence in layer 4 indicating the location of barrel cortex. The image was created by merging frames collected at 4x into an image of the entire section. The green fluorescence is entirely localized within the S1 barrel field. Blue fluorescence shows cell bodies stained with DAPI. This example injection site was the largest in our data set, and therefore the expression of eNpHR3.0 was within the limits of S1 barrel cortex in all mice.

(C) Quantification of the mediolateral extent of YFP fluorescence near the center of the injection sites, expressed as the FWHM (full width at half maximum) based on Gaussian fits to the smoothed line profiles of green fluorescence through layer 2/3 of the barrel cortex (N = 4 mice). All viral injections were centered on the C2 barrel column, which lies in the middle of the posterior barrel cortex, and all injection sites were smaller than the ~1.5 mm extent of the barrel field, indicating that NpHR3.0 expression was restricted to S1.
